# Mechanisms of Perceived Treatment Assignment and Subsequent Expectancy Effects in a Double Blind Placebo Controlled RCT of Major Depression

**DOI:** 10.3389/fpsyt.2018.00424

**Published:** 2018-09-07

**Authors:** Johannes A. C. Laferton, Sagar Vijapura, Lee Baer, Alisabet J. Clain, Abigail Cooper, George Papakostas, Lawrence H. Price, Linda L. Carpenter, Audrey R. Tyrka, Maurizio Fava, David Mischoulon

**Affiliations:** ^1^Department of Psychiatry, Brigham and Women's Hospital, Harvard Medical School, Harvard University, Boston, MA, United States; ^2^Department of Clinical Psychology and Psychotherapy, Friedrich-Alexander-Universität Erlangen-Nürnberg, Erlangen, Germany; ^3^Department of Clinical Psychology and Psychotherapy, Psychologische Hochschule Berlin, Berlin, Germany; ^4^Depression Clinical and Research Program, Department of Psychiatry, Massachusetts General Hospital, Harvard Medical School, Boston, MA, United States; ^5^Mood Disorders Research Program, Laboratory for Clinical and Translational Neuroscience, Butler Hospital, Department of Psychiatry and Human Behavior, Alpert Medical School, Brown University, Providence, RI, United States

**Keywords:** major depressive disorder, SAMe, escitalopram, placebo, perceived treatment assignment, un-blinding, double blind randomized controlled trial, bias

## Abstract

**Objective:** It has been suggested that patients' perception of treatment assignment might serve to bias results of double blind randomized controlled trials (RCT). Most previous evidence on the effects of patients' perceptions and the mechanisms influencing these perceptions relies on cross-sectional associations. This re-analysis of a double blind, placebo controlled RCT of pharmacological treatment of major depression set out to gather longitudinal evidence on the mechanism and effects of patients' perceived treatment assignment in the pharmacological treatment of major depression.

**Methods:** One-hundred eighty-nine outpatients with DSM-IV diagnosed major depression were randomized to SAMe 1,600–3,200 mg/d, escitalopram 10–20 mg/days, or placebo for 12 weeks. Data on depressive symptoms (17-item Hamilton Depression Scale; HDRS-17), adverse events and patients' perceived treatment assignment was collected at baseline, week 6, and week 12. The re-analysis focused on *N* = 166 (out of the originally included 189 participants) with available data on perceived treatment assignment.

**Results:** As in the parent trial, depressive symptoms (HDRS-17) significantly decreased over the course of 12 weeks and there was no difference between placebo, SAMe or escitalopram. A significant number of patients changed their perceptions about treatment assignment throughout the trial, especially between baseline and week 6. Improvement in depressive symptoms, but not adverse events significantly predicted perceived treatment assignment at week 6. In turn, perceived treatment assignment at week 6, but not actual treatment, predicted further improvement in depressive symptoms at week 12.

**Conclusions:** The current results provide longitudinal evidence that patients' perception of treatment assignment systematically change despite a double blind procedure and in turn might trigger expectancy effects with the potential to bias the validity of an RCT.

Parent study grant number: R01 AT001638 Parent study ClinicalTrials. gov Identifier: NCT00101452

## Introduction

The belief that one is taking a medication can lead to improvement in numerous health conditions regardless of the presence or absence of a pharmacologic agent (1, 2). This expectation effect is specifically pronounced in the pharmacological treatment of depression (3, 4). Double blind randomized controlled trials (RCTs) assume that these expectations are equally balanced across treatment arms. Yet, the effectiveness of blinding in RCTs is rarely assessed or reported, and there are suggestions in the literature that patients are frequently un-blinded (5–7). If patients do learn which treatment arm they are in, expectancy effects due to perceived treatment assignment are no longer controlled for. This introduces a considerable amount of bias, as meta-epidemiological studies typically find un-blinded studies to exaggerate effect size by more than 30% compared to blinded studies (8–10). Moreover, it is important to note that not only an actual un-blinding, but any between-groups imbalance of the perceived treatment assignment can bias the results of a trial (11, 12).

Possible mechanisms that may influence perceived treatment assignment include the physical characteristics of the medication and the placebo, medication side effects, or beneficial effects on the health condition (13). Regarding the former, taste, color, shape, size, route and process of administration (13) might lead to un-blinding, if they differ between drug and placebo. Moreover, medication side effects could inadvertently serve to influence perception of treatment assignment (14, 15). Studies have shown that side effects are associated with patients and independent evaluators guessing treatment assignment (15, 16). The experience of side effects could then increase the treatment effect by enhancing the patient's expectation of benefit. This possibility is supported by both experimental and clinical evidence. Thus, for example, in an experimental pain task (17), participants receiving a placebo that produces side effects (so called, “active placebo”) achieved higher pain thresholds than those who received a non-active placebo. Clinical trials on the pharmacological treatment of pain or depression using active placebos as a control condition have found smaller differences between active medication and the placebo arm compared to similar trials using non-active placebos (18–21). It should be noted that in order for physical characteristics or side effects to influence perceived treatment assignment, the participant needs at least a certain amount of knowledge about these characteristics of the drug. Finally, improvement of symptoms may also indicate participants' perception of treatment assignment. Previous studies among various health conditions have shown an association between clinical improvement and patient perceptions regarding treatment assignment (22–27). However, a major limitation of most of those analyses is that perceived treatment assignment was elicited either before or after treatment, making it impossible to investigate mechanisms of un-blinding and its prospective impact on treatment outcome. If assessed at the beginning of a trial, it cannot be concluded whether mechanisms such as side effects or health improvement have had an influence on the perception of treatment assignment. In contrast, assessment at the end of a study does not indicate whether more side effects or greater improvement in health were due to the perceived treatment assignment, or whether the perception of treatment assignment was due to experienced side effects or improvement in health. Experimental evidence suggests that experienced improvement influences perceived treatment assignment, which in turn influences treatment outcome (28). Whether this is true for clinical trials remains unclear so far. Moreover, when using a single time point assessment, one cannot determine whether participants change their perception of treatment assignment throughout a trial.

To better understand (1) whether the perception of the treatment assignment changes over the course of a study, (2) and whether these changes are influenced by side effects or health improvement, and (3) whether the perceived treatment assignment is prospectively related to the treatment effects, a re-analysis of a three-armed, double blind RCT on the treatment of major depression was conducted. The parent trial (29) examined the effects of escitalopram or S-adenosyl-L-methionine (SAMe) vs. placebo in patients with major depression and assessed perceived treatment assignment at several points throughout the trial. Moreover, in the parent study, no significant differences in improvement in the 17-item Hamilton Depression Rating Scale (HDRS-17) total score or response rates were found between the three treatment arms, making it particularly interesting to investigate expectancy effects due to possible bias in perceived treatment.

## Materials and methods

### Study design and procedure

This study is a secondary analysis of a two center, three-arm, double blind RCT (29) on the treatment of major depression with escitalopram or SAMe vs. placebo (clinical trials.gov: NCT00101452) conducted at two academic psychiatry centers in the U.S. Detailed methods for the parent trial have been described elsewhere (29). The study was approved by both local Institutional Review Boards.

### Participants

One-hundred eighty-nine outpatients, 18–80 years old, who met criteria for current major depressive episode according to Structured Clinical Interview for DSM-IV (30) plus screening and baseline scores of ≥25 on the Inventory of Depressive Symptomatology-Clinician-Rated (31), were recruited from April 2005 to December 2009 through clinician referral and general advertisement (e.g., “Have you lost interest in things you used to enjoy, had appetite or sleep changes? Are you interested in natural remedies? Participate in a research study of a naturally occurring supplement called SAMe in treating Major Depressive Disorder”) in local newspapers, radio, and television. A ≥ 6 week use of SAMe or escitalopram during the concurrent episode as well as severe medical or other primary psychiatric disorder were exclusion criteria [for detailed description of exclusion criteria see (29)].

### Procedure

After written informed consent participants were randomized in a 1:1:1 manner (stratified by center) for 12 weeks of double-blind treatment with SAMe (1,600 mg/d), escitalopram (10 mg/d), or placebo. A double-dummy design was used to maintain the blind, since SAMe tablets differed in appearance from escitalopram tablets. Participants were made aware of their odds of receiving any particular one of the three possible treatments. At week 6, for non-responders (<50% HAM-D reduction) escitalopram dose could be increased to 20 mg/d and SAMe to 3,200 mg/d for weeks 7–12. Participants who experienced intolerable side effects at the higher dose were allowed to decrease their dose to the previous level.

### Assessment

Assessment relevant for the reanalysis took place during baseline, visit 4 (week 6) and visit 7 (week 12; end of active treatment). Antidepressant efficacy was assessed with the Hamilton 17-item Depression Rating Scale [HDRS-17; (32)]. Side effects were assessed using the Systematic Assessment for Treatment Emergent Events-Systematic Inquiry [SAFTEE (33)]. Side effects documented on the SAFTEE were categorized by severity as 0 (none), 1 (mild), 2 (moderate), and 3 (severe). Scores were calculated based on the number of adverse events reported by each subject that were treatment-emergent, which we defined as any SAFTEE side effect for which severity increased by 1 or more levels from baseline. Besides an overall side effect score, sub-category scores for gastrointestinal and sexual functioning side effects were calculated based on known pharmacologic profiles of the active treatments and side effect patterns reported in the parent study (29). In order to assess perception of treatment assignment patients were asked to guess whether they believed to have received SAMe, escitalopram or placebo.

### Data analysis

This re-analysis focused on the acute treatment phase only (baseline-12 weeks) including *N* = *166* participants from the intent-to-treat sample, with at least one post-baseline visit and available data on perceived treatment assignment. Patients with missing data on perceived treatment assignment did not differ from those included in the analysis with regard to HRDS-17 scores and side effects at the respective time points.

Frequency distribution, means and standard deviations were assessed for each variable. Non-normally distributed variables were log_10_-transformed in order to satisfy the statistical assumptions of parametric tests. Baseline differences were tested with analysis of variance (ANOVA) for parametric data, and χ^2^-tests for categorical data. Change in clinical variables was analyzed with mixed ANOVAs with treatment assignment as the between-participant factor and time as the within-participants factor. Significant main or interaction effects were analyzed by Bonferroni corrected *post-hoc* tests. Differences in perceived treatment assignment distributions were analyzed using χ^2^-tests. To assess whether clinical improvement or side effects were prospectively associated with perceived treatment assignment, logistic regression analyses were performed using clinical improvement and side effects as predictors, actual treatment assignment (dummy coded with placebo as the reference condition) and previous perceived treatment assignment (active treatment vs. placebo) as covariates, and whether participants perceived themselves to be on active medication [placebo = 0; active treatment (SAMe or escitalopram) = 1] at the successive time point as the dependent variable. To assess whether perceived active treatment affected subsequent clinical improvement, linear multiple regression analysis were performed with perceived treatment [placebo = 0; active treatment (SAMe or escitalopram) = 1] as predictor, actual treatment assignment (dummy coded with placebo as the reference condition) and pre HDRS-17 score as covariates, and successive HDRS-17 score as dependent variable. To test for treatment arm specific effects of perceived treatment, the interaction term between actual treatment assignment and perceived treatment assignment was added to the multiple regression analysis and additional Bonferroni corrected *post-hoc* tests were carried out for each treatment arm individually. For all analyses, two-tailed significance was set at *p* < 0.05. All calculations were performed with SPSS Version 23 (Chicago, Illinois).

## Results

### Trial characteristics

Demographic and baseline clinical characteristics for each treatment group are reported in Table 1. No significant differences were observed between the treatment groups.

**Table 1:** Demographic and clinical characteristics throughout trial.

	**Placebo (*n* = 52)**	**SAMe (*n* = 59)**	**Escitalopram (*n* = 55)**	**Baseline differences**
Age years *M (SD)*	43.68 (16.51)	45.04 (14.16)	46.12 (13.47)	*F*_(2, 163)_ = 0.39, *p* = 0.674
Sex *f (%)* female	27 (51.9)	32 (54.2)	27 (49.1)	χ^2^(2) = 0.30; *p* = 0.682
Race *f (%)* Caucasian (*MD* = 15)	43 (84.3)	41 (82.0)	40 (80.0)	χ^2^(2) = 0.32; *p* = 0.852
Education *f (%)* college (*MD* = 8)	37 (72.5)	39 (70.9)	37 (71.2)	χ^2^(2) = 0.04; *p* = 0.980
Currently married/living with someone	14 (28.0)	10 (18.2)	15 (18.2)	χ^2^(2) = 2.02; *p* = 0.364
Employment *f (%)* working	13 (25.0)	28 (47.5)	20 (36.4)	χ^2^(2) = 1.49; *p* = 0.473
Dose increase at week 6 *f (%; MD* = 39)	29 (67.4)	20 (47.6)	27 (64.3)	χ^2^(2) = 3.99; *p* = 0.136
**DEPRESSIVE SYMPTOMS (HAMD)**				
Baseline *M (SD)*	19.44 (4.03)	19.12 (4.81)	19.71 (4.84)	Main effect time: *F*_(2, 93)_ = 79.58, *p* < 0.001
Week 6 *M* (*SD; MD* = 38)	13.42 (5.69)	11.09 (7.22)	13.22 (6.99)	Main effect group: *F*_(2, 94)_ = 0.79, *p* = 0.454
Week 12 *M* (*SD; MD* = 69)	12.00 (6.96)	11.12 (6.74)	10.78 (6.58)	Interaction group × time: *F*_(4, 94)_ = 1.34, *p* = 0.257
**SIDE EFFECTS (SAFTEE)**				
**Overall**			
Week 6 *M (SD; MD* = 40)	4.41 (4.48)	3.84 (3.56)	5.40 (4.42)	Main effect time: *F*_(1, 93)_ = 0.16, *p* = 0.690
Week 12 *M (SD; MD* = 70)	4.16 (3.62)	4.28 (4.15)	4.82 (4.29)	Main effect group: *F*_(2, 93)_ = 1.51, *p* = 0.227
				Interaction group × time: *F*_(2, 93)_ = 0.19, *p* = 0.191
**Gastrointestinal**				
Week 6 *M (SD; MD* = 40)	0.29 (0.51)	0.58 (0.91)	0.55 (0.86)	Main effect time: *F*_(1, 93)_ = 0.22, *p* = 0.644
Week 12 *M (SD; MD* = 70)	0.35 (0.61)	0.83 (1.23)	0.48 (0.74)	Main effect group: *F*_(2, 93)_ = 2.03, *p* = 0.137
				Interaction group × time: *F*_(2, 93)_ = 0.90, *p* = 0.409
**Sexual Function**				
Week 6 *M* (*SD; MD* = 40)	0.17 (0.54)	0.19 (0.59)	0.54 (0.89)	Main effect time: *F*_(1, 93)_ = 0.09,
Week 12 *M* (*SD; MD* = 70)	0.23 (0.56)	0.18 (0.58)	0.72 (0.96)	Main effect group: *F*_(2, 93)_ = 7.01,
				Interaction group × time: *F*_(2, 93)_ = 0.04, *p* = 0.964.

*MD, Missing Data*.

### Changes in depressive symptoms and side effects over time

Depressive symptoms significantly declined over time (see Table 1). However, there was no significant between-group difference or group × time interaction. For both total side effect score and gastrointestinal side effects (see Table 1), there was no significant change from week 6 to week 12, between-group difference, or group × time interaction. For sexual functioning side effects (see Table 1), there was no significant change from week 6 to week 12, or group × time interaction, but the between-group difference was significant. Bonferroni-corrected *post-hoc* tests revealed that sexual functioning side effects were significantly higher with escitalopram compared to SAMe (*p* = 0.002) and to placebo (*p* = 0.009), but not different between SAMe and placebo (*p* = 0.714).

### Changes in perceived treatment assignment distributions over time

**Figure 1:**
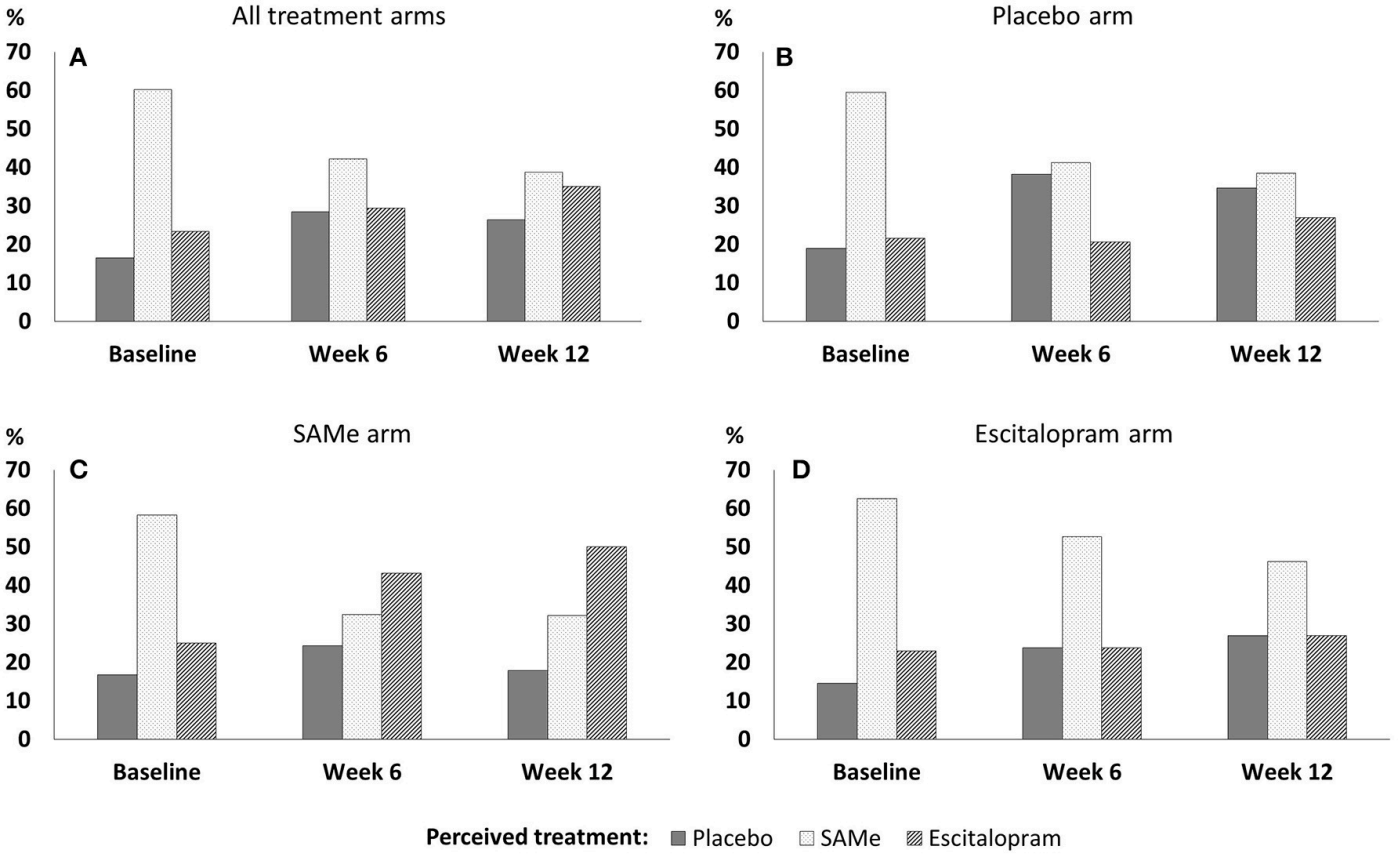
Perceived treatment assignment throughout the trial in **(A)** all treatment arms, **(B)** the placebo arm, **(C)** the SAMe arm, and **(D)** the escitalopram arm.

Participants' perceptions of treatment assignment throughout the study can be seen in Figure 1. At baseline (before application of treatment), participants in the SAMe group (χ^2^ = 14.00, *df* = 2, *p* = 0.001), the escitalopram group (χ^2^ = 18.88, *df* = 2, *p* < 0.001) and the placebo group (χ^2^ = 11.41, *df* = 2, *p* = 0.003) were expecting to receive SAMe significantly more often than would be expected, based on an equal assignment probability (1/3). At week 6, only participants in the escitalopram arm perceived themselves to be on SAMe more often than by chance (χ^2^ = 6.37, *df* = 2, *p* = 0.041). Participants' perceived treatment assignment in the SAMe (χ^2^ = 2.00, *df* = 2, *p* = 0.368) and placebo groups (χ^2^ = 2.53, *df* = 2, *p* = 0.282) did not significantly differ from that expected in an equal treatment distribution.

At week 12, participants' perceived treatment assignment did not significantly differ from an equal distribution in the SAMe arm (χ^2^ = 4.36, *df* = 2, *p* = 0.113), the escitalopram arm (χ^2^ = 1.92, *df* = 2, *p* = 0.283) or the placebo arm (χ^2^ = 0.54, *df* = 2, *p* = 0.764). There was no association between participants' baseline perceived treatment assignment and drop out by week 6 (χ^2^ = 0.04, *df* = 2, *p* = 0.982) and week 6 perceived treatment assignment and drop out by week 12 (χ^2^ = 1.90, *df* = 2, *p* = 0.387).

Overall, almost twice as many patients changed their perceived treatment assignment between baseline and week 6 (48.9%), compared to between week 6 and week 12 (26.0%; see Table 2 for detailed within-person change patterns). Between baseline and week 6 patients in the placebo group changed their perceived treatment assignment more often (60%) than patients in the SAMe and the escitalopram group (42.4%; 48.6%). Between week 6 and week 12 patients in the SAMe group changed their perception of treatment assignment more frequently (38.8%) than in the escitalopram and the placebo group (both: 20.8%).

**Table 2:** Changes in perceived treatment assignment from baseline to week 6 and week 6 to week 12 by actual treatment group.

	**Change baseline to week 6**	**Change week 6 to week 12**
	**f (%)**	**f (%)**
**Active medication = PBO**	***n* = 25**	***n* = 24**
no change	10 (40.0)	19 (79.2)
SAMe to ESC	4 (16.0)	1 (4.2)
SAMe to PBO	5 (20.0)	1 (4.2)
ESC to SAMe	2 (8.0)	0 (0.0)
ESC to PBO	2 (8.0)	0 (0.0)
PBO to SAMe	1 (4.0)	2 (8.3)
PBO to ESC	1 (4.0)	1 (4.2)
**Active medication** = **SAMe**	***n*** = **32**	***n*** = **25**
no change	19 (57.6)	16 (61.5)
SAMe to ESC	6 (18.2)	4 (15.4)
SAMe to PBO	2 (6.1)	1 (3.8)
ESC to SAMe	1 (3.0)	2 (7.7)
ESC to PBO	1 (3.0)	1 (3.8)
PBO to SAMe	2 (6.1)	2 (7.7)
PBO to ESC	2 (6.1)	0 (0.0)
**Active medication** = **ESC**	***n*** = **35**	***n*** = **24**
no change	18 (51.4)	19 (79.2)
SAMe to ESC	4 (11.4)	2 (8.3)
SAMe to PBO	6 (17.7)	0 (0.0)
ESCto SAMe	4 (11.4)	1 (4.2)
ESC to PBO	0 (0.0)	1 (4.2)
PBO to SAMe	2 (5.7)	1 (4.2)
PBO to ESC	1 (2.9)	0 (0.0)

*PBO, placebo; SAMe, S-adenosyl-L-methionine; ESC, escitalopram*.

### Factors associated with perceived treatment assignment

Prospective associations of clinical improvement and side effects with whether participants perceived to be on an active medication (SAMe or escitalopram) or placebo are reported in Table 3. At week 6, participants were significantly more likely to perceive that they were receiving active medication if they experienced clinical improvement. Among participants who experienced a reduction in HDRS-17 score of 13 or greater, all perceived that they were assigned to an active medication group. There was no threshold below which participants would certainly perceive themselves to be on placebo. Side effects and actual treatment assignment were not associated with participants' perception that they were receiving an active medication at week 6. Clinical improvement between week 6 and week 12, side effects, and actual treatment assignment, were not associated with participants' perceived treatment assignment at week 12.

**Table 3:** Improvement and adverse effects predicting patients' perception to be on active medication (SAMe or escitalopram) or placebo at week 6 and week 12 using logistic regression analysis.

	***B (SE)***	***p***	***OR (95%−CI)***
**PERCEIVED TREATMENT ASSIGNMENT AT WEEK 6 (*****n*** = **92)**			
Improvement (Baseline – week 6; HDRS-17)	0.21 (0.06)	0.001	1.23 (1.09; 1.39)
**Side Effects Week 6 (SAFTEE)**			
Overall	−0.06 (1.15)	0.957	0.94 (0.09; 8.92)
Gastrointestinal	1.26 (1.95)	0.519	3.52 (0.07; 161.09)
Sexual function	−2.42 (1.72)	0.160	0.09 (0.01; 2.59)
**Drug Assignment**			
SAMe (vs. placebo)	0.84 (0.75)	0.266	2.31 (0.53; 10.10)
Escitalopram (vs. placebo)	1.00 (0.72)	0.165	2.73 (0.66; 11.25)
**Baseline Perceived Treatment Assignment**			
Active medication (vs. placebo)	1.41 (0.73)	0.054	4.10 (0.98; 17.23)
Constant	1.44 (1.11)	0.193	0.236
*R^2^* = 0.28 (Cox & Snell) 0.42 (Nagelkerke) χ^2^_(7)_ = 30.66, *p* < 0.001			
**PERCEIVED TREATMENT ASSIGNMENT AT WEEK 12 (*****n*** = **71)**			
Improvement (week 6–week 12; HDRS-17)	−0.03 (0.06)	0.653	0.97 (0.86; 1.10)
**Side Effects Week 12 (SAFTEE)**			
Overall	−1.35 (1.63)	0.408	0.26 (0.01; 6.36)
Gastrointestinal	1.88 (2.23)	0.400	7.12 (0.09; 515.08)
Sexual function	−1.18 (2.36)	0.617	0.37 (0.00; 31.27)
**Drug Assignment**			
SAMe (vs. placebo)	0.00 (0.95)	0.998	1.00 (0.16; 6.41)
Escitalopram (vs. placebo)	−0.02 (0.95)	0.980	0.98 (0.15; 6.29)
**Week 6 Perceived Treatment Assignment**			
Active medication (vs. placebo)	3.25 (0.75)	<0.001	25.89 (5.91; 113.31)
Constant	−0.03 (1.12)	0.975	0.966
*R^2^* = 0.36 (Cox & Snell) 0.52 (Nagelkerke) χ^2^(7) = 31.32, *p* < 0.001			

*The predictors drug assignment and perceived treatment assignment were dummy coded with placebo as reference condition*.

### Prospective associations of perceived treatment assignment and subsequent improvement

**Figure 2:**
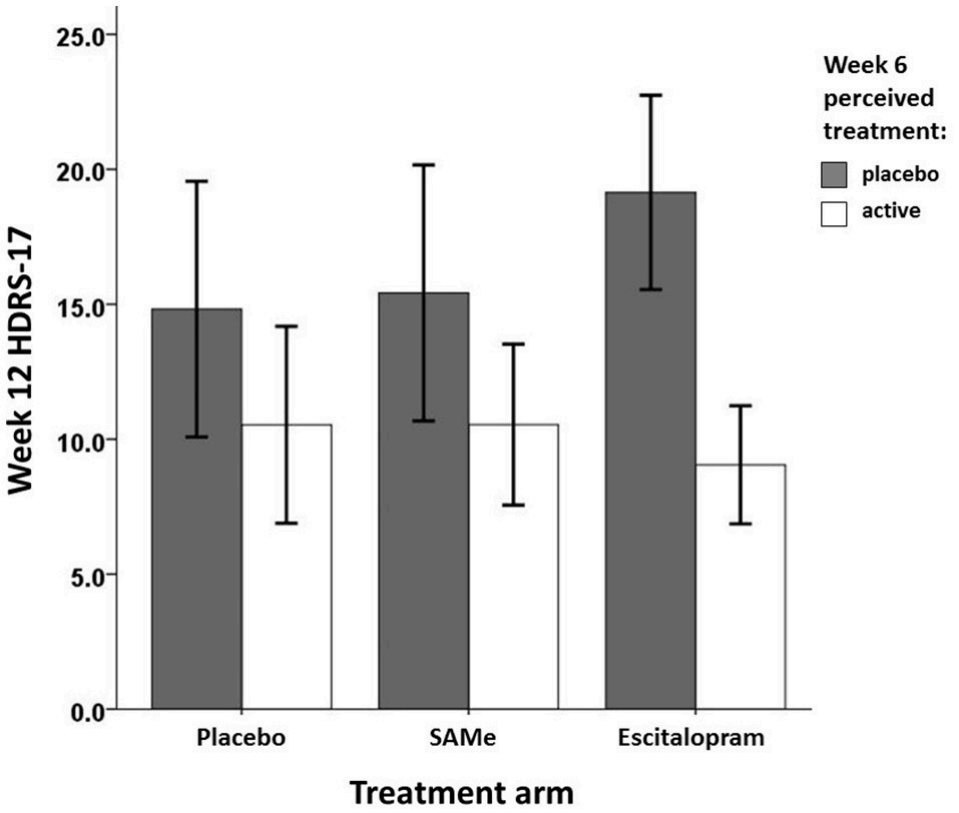
Mean week 12 treatment outcome by week 6 perceived treatment and actual treatment assignment.

Prospective associations of participants' perceived treatment assignment (active vs. placebo) and actual treatment assignment on clinical improvement are shown in Table 4. Controlling for baseline HDRS-17, neither actual nor perceived treatment assignment predicted HDRS-17 scores at week 6. However, controlling for week 6 HDRS-17, patients' perceived treatment assignment at week 6, but not actual treatment assignment predicted week 12 HDRS-17 scores. Participants perceiving they were taking active medication at week 6 showed significantly higher improvement in depressive symptoms at week 12 than participants believing they were taking placebo. *Post-hoc* analysis revealed no significant interaction between actual treatment assignment and week 6 perceived treatment predicting HDRS-17 improvement at week 12 [*R*^2^ = 0.02, *F*_(2, 76)_ = 1.28*, p* = 0.284]. However, Bonferroni corrected *post-hoc* sub group analyses of the effect of perceived treatment at week 6 on treatment outcome at week 12 within each treatment group individually (Table 5) indicated that the effect of perceived treatment assignment was significant in the escitalopram treatment arm but not in the placebo and SAMe treatment arm (Figure 2).

**Table 4:** Longitudinal associations of patients' perceived treatment assignment and actual drug assignment with subsequent improvement in depression (HDRS-17).

	**ß**	***B [CI]***	***SE (B)***	***t***	***p***
**WEEK 6 HDRS-17 (*****n*** = **104)**					
Baseline HDRS-17	0.28	0.41 [0.14; 0.69]	0.14	2.97	0.004
**Drug Assignment**					
SAMe (vs. placebo)	−0.21	−2.92 [−6.03; 0.19]	1.57	1.86	0.065
Escitalopram (vs. placebo)	−0.03	−0.39 [−3.54; 2.67]	1.57	0.25	0.802
Week 6 perceived treatment assignment = active (vs. placebo)	−0.07	−1.32 [−4.69; 2.05]	1.69	0.78	0.440
*R^2^* = 0.11, *F*_4, 99_ = 3.71, *p* = 0.007					
**WEEK 12 HDRS-17 (*****n*** = **83)**					
Week 6 HDRS-17	0.47	0.47 [0.26; 0.68]	0.11	4.40	<0.001
**Drug Assignment**					
SAMe (vs. placebo)	0.09	1.21 [−1.77; 4.19]	1.49	0.81	0.420
Escitalopram (vs. placebo)	−0.06	−0.82 [−3.88; 2.27]	1.54	0.51	0.605
Week 6 perceived treatment assignment = active (vs. placebo)	−0.22	−3.10 [−6.12; −0.09]	1.51	2.05	0.044
*R^2^* = 0.13, *F_(4, 78)_* = 10.41, *p* < 0.001					

*The predictors drug assignment and perceived treatment assignment were dummy coded with placebo as reference condition*.

**Table 5:** Longitudinal associations of patients' perceived treatment assignment with subsequent improvement in depression (HDRS-17) at week 12 individually by treatment group.

	**ß**	***B [CI]***	***SE (B)***	***t***	***p***
**ACTUAL TREATMENT** = **PLACEBO (*****n*** = **26)**					
Week 6 HDRS-17	0.09	0.15 [−0.42; 0.65]	0.26	0.45	0.999
Week 6 perceived treatment assignment = active (vs. placebo)	−0.27	−3.80 [−9.89; 2.93]	2.95	1.29	0.630
*R^2^* = 0.10, *F*_(2, 23)_ = 1.32, *p* = 0.286					
**ACTUAL TREATMENT** = **SAMe (*****n*** = **30)**					
Week 6 HDRS-17	0.65	0.60 [0.28; 0.92]	0.16	3.89	0.003
Week 6 perceived treatment assignment = active (vs. placebo)	0.00	−0.00 [−5.39; 5.93]	2.63	0.00	0.999
*R^2^* = 0.42, *F*_(1, 28)_ = 9.80, *p* = 0.001					
**ACTUAL TREATMENT** = **ESCITALOPRAM (*****n*** = **27)**					
Week 6 HDRS-17	0.58	0.59 [0.32; 0.87]	0.13	4.42	<0.010
Week 6 perceived treatment assignment = active (vs. placebo)	−0.37	−5.18 [−9.03; −1.33]	1.87	2.78	0.030
*R^2^* = 0.73, *F*_(2, 24)_ = 32.46, *p* < 0.001					

*Perceived treatment assignment was dummy coded with placebo as reference condition. p-values have been adjusted for multiple testing using Bonferroni correction*.

## Discussion

This re-analysis of a three arm, two-center, double blind RCT of SAMe or escitalopram vs. placebo for the treatment of major depression shows that a significant number of patients did change their perception of treatment assignment throughout the trial, corroborating previous analyses on longitudinal changes of perceived treatment assignment in clinical trials (11, 23). The large majority of change in perceived treatment assignment happened throughout the first 6 weeks of treatment and was significantly predicted by the preceding improvement in depressive symptomatology. Side effects did not seem to have influenced perceived treatment assignment. Although side effects are frequently mentioned as a potential mechanism informing perceived treatment assignment, this result is consistent with another re-analysis of an RCT containing two active treatments for opioid dependency. Oviedo-Joekes et al. (34) found that desired drug effects (drug related highs) but not overall side effects were associated with perceived treatment allocation. Possibly, in trials with more than one active treatment, side effects might not be a pivotal mechanism influencing perceived treatment assignment, since it may be more difficult to guess between two active treatments based simply on side effects. Moreover, patients randomized to placebo in the current re-analysis did not differ from the active treatment groups in terms of side-effects indicating a nocebo effect (35, 36). Hence, variance in side effects might not have been heterogeneous enough to be associated with perceived treatment. While a majority of patients expected to receive SAMe before treatment began, there was no imbalance of perceived group allocation in favor of actual group allocation during the active treatment phase. This indicates that patients overall were successfully blinded regarding their specific treatment allocation until the end the treatment phase.

However, patients believing to be on active medication at week 6 showed significantly higher improvement in depressive symptoms at week 12 than patients believing to be on placebo, indicating expectancy effects due to perceived treatment assignment. The expectancy effect did not seem to be influenced by selective drop out, given the lack of association between perceived treatment and subsequent attrition. It remains unclear whether the expectancy effect was of the same or different magnitude among all treatment arms. *Post-hoc* analysis suggests that this effect might have been more pronounced in the escitalopram treatment arm. However, the omnibus test for an interaction between perceived and actual treatment was not significant. Therefore, this suggestion remains speculative for the trial at hand and should encourage better powered re-analyses to further explore this issue.

Notwithstanding, while most previous studies—due to their cross-sectional analysis—were unable to differentiate whether the perception of receiving active medication enhanced the treatment response via expectancy effects, or whether the improvement at the end of these trials influenced the final perception of treatment assignment, this re-analysis indicates that both might be true successively. The suggested pathway of expectancy effects due to perceived treatment assignment found in experimental research (28)–improvement influences perceived treatment, which in turn influences treatment outcome-appears to be validated within this re-analysis of a clinical trial.

In view of the above, this re-analysis now longitudinally confirms expectancy effects in double blind RCTs, and further ads to research highlighting important limitations for the interpretation of effects found in double blind placebo controlled RCTs. In clinical practice, patients do not have reason to doubt that they are receiving active medication. In placebo controlled RCTs, however, this doubt is justified and potentially induces decreased expectancy, which results in an underestimation of the effectiveness of a drug compared to routine clinical practice (37, 38). More generally, double blind RCTs evaluate the specific treatment effect of a pharmacologic verum by comparing it to the response generated by the unspecific treatment effect in the placebo group. If the verum group's treatment response exceeds that in the placebo group, the drug is considered to have drug specific effects. However, such a judgment is only justifiable under the assumption that the treatment response in the verum group consists of the equivalent unspecific effects observed in the placebo group plus the specific effects of the verum (“additive model”). Such an additive model of drug and placebo effects, however, has frequently been questioned theoretically and empirically (39–41). In fact evidence from clinical trials and both behavioral and neuro-physiological experiments suggest that drug specific and unspecific effects can interact (38, 39) and hence might not be equal between a verum and a placebo arm. Therefore, drug specific effects can be both over and underestimated in double blind RCTs. Although results regarding differential expectancy effects with the treatment arms are inconclusive in the current re-analysis, based on the results of this RCT, it cannot be said with absolute certainty that neither SAMe nor escitalopram do have or do not have drug specific efficacy. There is the possibility that specific characteristics of the SAMe or esctitalopram arm induced expectancy effects, which would have led to an overestimation of drug specific effects. On the other hand, RCTs with more than one active treatment arm have been found to show enhanced expectancy effects since the uncertainty of receiving active treatment is lower (38). As a result, this might have led to ceiling effects masking the drug specific expectancy. To circumvent these pitfalls, new study designs to better evaluate the effects of pharmacological treatment have been proposed; these include the balanced placebo design, the balanced cross over design, balanced open-hidden design or delayed response design [see 39, 40 for more details]. However, until such innovations for testing pharmacological interventions become more established, double blind RCTs should at least assess and test for expectancy effects in a systematic manner.

Concerning the assessment of expectancy effects, the results of this re-analysis suggest that the current practice of measuring perceived treatment assignment only once—either at the beginning or at the end of a trial—is questionable. The disadvantages of end of trial assessment of perceived treatment assignment have already been mentioned above. However, some previous studies used baseline or early assessment of perceived treatment allocation for their analysis of blinding or expectancy effects. Yet, in this re-analysis the perception of treatment assignment at the start of the trial did not at all reflect the perception of treatment assignment throughout the trial, making it a very unreliable measure to operationalize un-blinding or expectancy effects. Future trials should use repeated assessment of patients' perceived treatment assignment to determine un-blinding and evaluate expectancy effects. In addition, it would be even more advisable to assess patients' outcome expectations throughout a trial. First, a patient who believes to be on a specific active medication but does not believe the medication to be effective will most likely not have any expectancy effects (37), a case that would not be differentiated by only assessing perceived treatment assignment. Secondly, expectations can be assessed on parametric (or at least ordinal) scales, giving the advantage of statistical power over the assessment of perceived treatment assignment assessed as nominal data. For further details on the assessment of patients' expectations in medical treatment see (42).

While not the focus of this re-analysis, one additional finding is of interest: a large majority of patients before the start of treatment expected to receive SAMe, despite being informed about the equal assignment probability. A similar pattern in favor of the “new” treatment has been observed in other trials before (11, 34). This might shed interest on the role of drug trial advertisement. For the trial of this re-analysis, advertisements emphasized the treatment of depression with SAMe, because it was thought that this would attract more participants interested in taking a natural product; this could have influenced patients' expectations. While the current re-analysis did not find any indication that these expectations influenced treatment effects, one might see some indication of advertisement or novelty effect (14) in the newest net-work meta-analysis on anti-depressants (43). Cipriani et al. did find that the same anti-depressant had a positive pooled effect size in trials when evaluated as a new agent, and negative effect sizes when evaluated as the “old” comparator agent. Hence, in trials with two or more active comparators it might be worthwhile to investigate whether framing such as “new” vs. “old” toward participants is an additional source of bias. If such a “novelty effect” existed, this would pose further evidence against an additive model.

Some limitations have to be considered when interpreting the results presented. First, the external validity is limited by the fact that the re-analysis was based on a three arm RCT with SAMe and escitalopram as the active treatments. As discussed above expectancy effects are considered to be higher in a study with two active treatments. Generalization to clinical practice is limited since patients usually have no doubt about receiving active medication. However, patients in clinical practice are likely to have expectations regarding the efficacy of the medication or expectations about side effects (42), which might also serve to influence treatment effects. Additionally, both active treatments are reasonably well tolerated. Therefore, it remains unclear, whether results would be different among active treatment with stronger side effect profiles (e.g., tricyclic antidepressants). Moreover, it remains unclear whether comparing a “classical” anti-depressant with a natural supplement anti-depressant might have different mechanisms of perceived treatment than in other trials. Related to that, it is unclear whether trial advertisement attracted individuals with specific interest in natural remedies, who might for example have more negative expectations about “classical” anti-depressants. Further, although there was no difference in participants providing perceived treatment data regarding depressive symptoms and side effects, nor an association with drop-out, one can not completely rule out whether missing guess data might be a source of bias.

In conclusion, this re-analysis showed that patients' perceptions about treatment assignment do change throughout a trial, that these perceptions appeared to be influenced by preceding improvement in depressive symptoms, and that perceptions about treatment assignment predicted further improvement. Building on previous cross-sectional and experimental evidence the current results further highlight issues with the interpretation of effects found in double blind RCTs. Future RCTs should apply multiple assessment of perceived treatment assignment and/or expectations throughout the trial. This would permit testing for possible expectation effects that might bias the comparison for specific efficacy, and provide the opportunity to further explore mechanisms of bias in double blind RCTs. Moreover, new study designs for testing pharmacological interventions should be considered, to get a more concise estimate of the specific effects of a pharmacological treatment.

## Ethics statement

This study was carried out in accordance with the recommendations of the U.S. Food and Drug Administration (FDA) guidelines with written informed consent from all subjects. All subjects gave written informed consent in accordance with the Declaration of Helsinki. The protocol was approved by the Institutional Review Boards of the Massachusetts General Hospital in Boston, MA and the Butler Hospital in Providence, RI.

## Data availability statement

The datasets for this manuscript are not publicly available yet, because the principal investigators are still in the process of actively analyzing the data and preparing manuscripts for publication, based on the main goals and outcome measures put forth in the original funded grant proposal. Requests to access the datasets should be directed to David Mischoulon, MD, Ph.D., dmischoulon@mgh.harvard.edu.

## Author contributions

JL, SV, and DM designed the re-analysis, conducted data analysis, and wrote the current manuscript. DM, LP, LC, AT, and MF designed and conducted the original randomized controlled trial this re-analysis is based on. LB and AJC assisted with the statistical analysis. GP and AC assisted with the writing of the manuscript.

### Conflict of interest statement

JL was supported by a fellowship within the Postdoc-Program of the German Academic Exchange Service (DAAD).

LP has received research support from Neuronetics, NIH, HRSA, and NeoSync. He has served on advisory panels for Abbott and AstraZeneca. He has served as a consultant to Gerson Lehrman, Wiley, Springer, Qatar National Research Fund, and Abbott.

LC has received research support from Neuronetics, NIH, and NeoSync. She has served on advisory panels or provided consulting services for Abbott, Corcept, Johnson &Johnson, and Takeda-Lundbeck.

AT has received research support from Neuronetics, NeoSync, and NIH.

GP has served as a consultant for Abbott Laboratories, Acadia Pharmaceuticals, Inc^*^, Alkermes, Inc, AstraZeneca PLC, Avanir Pharmaceuticals, Axsome Therapeutics^*^, Boston Pharmaceuticals, Inc., Brainsway Ltd, Bristol-Myers Squibb Company, Cephalon Inc., Dey Pharma, L.P., Eli Lilly Co., Genentech, Inc^*^, Genomind, Inc^*^, GlaxoSmithKline, Evotec AG, H. Lundbeck A/S, Inflabloc Pharmaceuticals, Janssen Global Services LLC^*^, Jazz Pharmaceuticals, Johnson & Johnson Companies^*^, Methylation Sciences Inc, Mylan Inc^*^, Novartis Pharma AG, One Carbon Therapeutics, Inc^*^, Osmotica Pharmaceutical Corp.^*^, Otsuka Pharmaceuticals, PAMLAB LLC, Pfizer Inc., Pierre Fabre Laboratories, Ridge Diagnostics (formerly known as Precision Human Biolaboratories), Shire Pharmaceuticals, Sunovion Pharmaceuticals, Taisho Pharmaceutical Co, Ltd, Takeda Pharmaceutical Company LTD, Theracos, Inc., and Wyeth, Inc.

GP has received honoraria (for lectures or consultancy) from Abbott Laboratories, Acadia Pharmaceuticals Inc, Alkermes Inc, Asopharma America Cntral Y Caribe, Astra Zeneca PLC, Avanir Pharmaceuticals, Bristol-Myers Squibb Company, Brainsway Ltd, Cephalon Inc., Dey Pharma, L.P., Eli Lilly Co., Evotec AG, Forest Pharmaceuticals, GlaxoSmithKline, Inflabloc Pharmaceuticals, Grunbiotics Pty LTD, Jazz Pharmaceuticals, H. Lundbeck A/S, Medichem Pharmaceuticals, Inc, Meiji Seika Pharma Co. Ltd, Novartis Pharma AG, Otsuka Pharmaceuticals, PAMLAB LLC, Pfizer, Pharma Trade SAS, Pierre Fabre Laboratories, Ridge Diagnostics, Shire Pharmaceuticals, Sunovion Pharmaceuticals, Takeda Pharmaceutical Company LTD, Theracos, Inc., Titan Pharmaceuticals, and Wyeth Inc.

GP has received research support (paid to hospital) from AstraZeneca PLC, Bristol-Myers Squibb Company, Forest Pharmaceuticals, the National Institute of Mental Health, Neuralstem, Inc, PAMLAB LLC, Pfizer Inc., Ridge Diagnostics (formerly known as Precision Human Biolaboratories), Sunovion Pharmaceuticals, Tal Medical, and Theracos, Inc.

GP has served (not currently) on the speaker's bureau for BristolMyersSquibb Co and Pfizer, Inc.

*Asterisk denotes activity undertaken on behalf of Massachusetts General Hospital.

MF: All disclosures can be view on line at: http://mghcme.org/faculty/faculty-detail/maurizio_fava

**Research Support**: Abbott Laboratories; Acadia Pharmaceuticals; Alkermes, Inc.; American Cyanamid;Aspect Medical Systems; AstraZeneca; Avanir Pharmaceuticals; AXSOME Therapeutics; Biohaven; BioResearch; BrainCells Inc.; Bristol-Myers Squibb; CeNeRx BioPharma; Cephalon; Cerecor; Clintara, LLC; Covance; Covidien; Eli Lilly and Company;EnVivo Pharmaceuticals, Inc.; Euthymics Bioscience, Inc.; Forest Pharmaceuticals, Inc.; FORUM Pharmaceuticals; Ganeden Biotech, Inc.; GlaxoSmithKline; Harvard Clinical Research Institute; Hoffman-LaRoche; Icon Clinical Research; i3 Innovus/Ingenix; Janssen R&D, LLC; Jed Foundation; Johnson & Johnson Pharmaceutical Research & Development; Lichtwer Pharma GmbH; Lorex Pharmaceuticals; Lundbeck Inc.; Marinus Pharmaceuticals; MedAvante; Methylation Sciences Inc; National Alliance for Research on Schizophrenia & Depression (NARSAD); National Center for Complementary and Alternative Medicine (NCCAM);National Coordinating Center for Integrated Medicine (NiiCM); National Institute of Drug Abuse (NIDA); National Institute of Mental Health (NIMH); Neuralstem, Inc.; NeuroRx; Novartis AG; Organon Pharmaceuticals; Otsuka Pharmaceutical Development, Inc.; PamLab, LLC.; Pfizer Inc.; Pharmacia-Upjohn; Pharmaceutical Research Associates., Inc.; Pharmavite&174; LLC; PharmoRx Therapeutics; Photothera; Reckitt Benckiser; Roche Pharmaceuticals; RCT Logic, LLC (formerly Clinical Trials Solutions, LLC); Sanofi-Aventis US LLC; Shire; Solvay Pharmaceuticals, Inc.; Stanley Medical Research Institute (SMRI); Synthelabo; Taisho Pharmaceuticals; Takeda Pharmaceuticals; Tal Medical; VistaGen; Wyeth-Ayerst Laboratories

**Advisory Board/Consultant**: Abbott Laboratories; Acadia; Affectis Pharmaceuticals AG; Alkermes, Inc.; Amarin Pharma Inc.; Aspect Medical Systems; AstraZeneca; Auspex Pharmaceuticals; Avanir Pharmaceuticals; AXSOME Therapeutics; Bayer AG; Best Practice Project Management, Inc.; Biogen; BioMarin Pharmaceuticals, Inc.; Biovail Corporation; BrainCells Inc; Bristol-Myers Squibb; CeNeRx BioPharma; Cephalon, Inc.; Cerecor; CNS Response, Inc.; Compellis Pharmaceuticals; Cypress Pharmaceutical, Inc.; DiagnoSearch Life Sciences (P) Ltd.; Dinippon Sumitomo Pharma Co. Inc.; Dov Pharmaceuticals, Inc.; Edgemont Pharmaceuticals, Inc.; Eisai Inc.; Eli Lilly and Company; EnVivo Pharmaceuticals, Inc.; ePharmaSolutions; EPIX Pharmaceuticals, Inc.; Euthymics Bioscience, Inc.; Fabre-Kramer Pharmaceuticals, Inc.; Forest Pharmaceuticals, Inc.; Forum Pharmaceuticals; GenOmind, LLC; GlaxoSmithKline; Grunenthal GmbH; Indivior; i3 Innovus/Ingenis; Intracellular; Janssen Pharmaceutica; Jazz Pharmaceuticals, Inc.; Johnson & Johnson Pharmaceutical Research & Development, LLC; Knoll Pharmaceuticals Corp.; Labopharm Inc.; Lorex Pharmaceuticals; Lundbeck Inc.; Marinus Pharmaceuticals; MedAvante, Inc.; Merck & Co., Inc.; MSI Methylation Sciences, Inc.; Naurex, Inc.; Navitor Pharmaceuticals, Inc.; Nestle Health Sciences; Neuralstem, Inc.; Neuronetics, Inc.; NextWave Pharmaceuticals; Novartis AG; Nutrition 21; Orexigen Therapeutics, Inc.; Organon Pharmaceuticals; Osmotica; Otsuka Pharmaceuticals; Pamlab, LLC.; Pfizer Inc.; PharmaStar; Pharmavite® LLC.; PharmoRx Therapeutics; Precision Human Biolaboratory; Prexa Pharmaceuticals, Inc.; PPD; Purdue Pharma; Puretech Ventures; PsychoGenics; Psylin Neurosciences, Inc.; RCT Logic, LLC (formerly Clinical Trials Solutions, LLC); Relmada Therapeutics, Inc.; Rexahn Pharmaceuticals, Inc.; Ridge Diagnostics, Inc.; Roche; Sanofi-Aventis US LLC.; Sepracor Inc.; Servier Laboratories; Schering-Plough Corporation; Shenox Pharmaceuticals; Solvay Pharmaceuticals, Inc.; Somaxon Pharmaceuticals, Inc.; Somerset Pharmaceuticals, Inc.; Sunovion Pharmaceuticals; Supernus Pharmaceuticals, Inc.; Synthelabo; Taisho Pharmaceuticals; Takeda Pharmaceutical Company Limited; Tal Medical, Inc.; Tetragenex; Teva Pharmaceuticals; TransForm Pharmaceuticals, Inc.; Transcept Pharmaceuticals, Inc.; Usona Institute,Inc.; Vanda Pharmaceuticals, Inc.; Versant Venture Management, LLC; VistaGen

**Speaking/Publishing**: Adamed, Co; Advanced Meeting Partners; American Psychiatric Association; American Society of Clinical Psychopharmacology; AstraZeneca; Belvoir Media Group; Boehringer Ingelheim GmbH; Bristol-Myers Squibb; Cephalon, Inc.; CME Institute/Physicians Postgraduate Press, Inc.; Eli Lilly and Company; Forest Pharmaceuticals, Inc.; GlaxoSmithKline; Imedex, LLC; MGH Psychiatry Academy/Primedia; MGH Psychiatry Academy/Reed Elsevier; Novartis AG; Organon Pharmaceuticals; Pfizer Inc.; PharmaStar; United BioSource, Corp.; Wyeth-Ayerst Laboratories.

**Stock/Other Financial Options**: Equity Holdings: Compellis; PsyBrain, Inc.

Royalty/patent, other income: Patents for Sequential Parallel Comparison Design (SPCD), licensed by MGH to Pharmaceutical Product Development, LLC (PPD) (US_7840419, US_7647235, US_7983936, US_8145504, US_8145505); and patent application for a combination of Ketamine plus Scopolamine in Major Depressive Disorder (MDD), licensed by MGH to Biohaven. Patents for pharmacogenomics of Depression Treatment with Folate (US_9546401, US_9540691).

Copyright for the MGH Cognitive & Physical Functioning Questionnaire (CPFQ), Sexual Functioning Inventory (SFI), Antidepressant Treatment Response Questionnaire (ATRQ), Discontinuation-Emergent Signs & Symptoms (DESS), Symptoms of Depression Questionnaire (SDQ), and SAFER; Lippincott, Williams & Wilkins; Wolkers Kluwer; World Scientific Publishing Co.Pte.Ltd.

DM has received research support from the Bowman Family Foundation, Nordic Naturals, and PharmoRx. He has provided unpaid consulting for Pharmavite LLC and Gnosis USA, Inc. He has received writing honoraria from Pamlab and Nordic Naturals, and speaking honoraria from Blackmores, and the MGH Psychiatry Academy. He has received royalties from Lippincott Williams & Wilkins, for textbook “Natural Medications for Psychiatric Disorders: Considering the Alternatives” (DM and Jerrold F Rosenbaum, Eds.).

The remaining authors declare that the research was conducted in the absence of any commercial or financial relationships that could be construed as a potential conflict of interest.
